# Comparison of the Differences in State-Trait Anxiety Inventory Scores and Insomnia Histories between Monozygotic and Dizygotic Twins: A Cross-Sectional Study Using KoGES HTS Data

**DOI:** 10.3390/jcm11144011

**Published:** 2022-07-11

**Authors:** So Young Kim, Dae Myoung Yoo, Mi Jung Kwon, Ji Hee Kim, Joo-Hee Kim, Woo Jin Bang, Hyo Geun Choi

**Affiliations:** 1 Bundang CHA Medical Center, Department of Otorhinolaryngology-Head and Neck Surgery, CHA University, Seongnam 13488, Korea; sossi81@hanmail.net; 2 Hallym Data Science Laboratory, Hallym University College of Medicine, Anyang 14066, Korea; ydm1285@naver.com; 3 Department of Pathology, Hallym Sacred Heart Hospital, Hallym University College of Medicine, Anyang 14068, Korea; mulank@hanmail.net; 4 Department of Neurosurgery, Hallym University College of Medicine, Anyang 14068, Korea; kimjihee.ns@gmail.com; 5 Department of Medicine, Division of Pulmonary, Allergy, and Critical Care Medicine, Hallym Sacred Heart Hospital, Hallym University College of Medicine, Anyang 14068, Korea; luxjhee@gmail.com; 6 Department of Urology, Hallym Sacred Heart Hospital, Hallym University College of Medicine, Anyang 14068, Korea; yybbang@gmail.com; 7 Department of Otorhinolaryngology-Head and Neck Surgery, Hallym University College of Medicine, Anyang 14068, Korea

**Keywords:** anxiety, insomnia, twin, cohort studies, epidemiology

## Abstract

The heritability of anxiety and its association with insomnia have been suggested. This study investigated the coincidence of anxiety and insomnia in monozygotic twins compared to dizygotic twins. The Korean Genome and Epidemiology Study 2005–2014 was used. The ≥20-year-old cohort population was composed of 1300 twin participants. A total of 980 monozygotic twins and 232 dizygotic twins were compared for the concordance for the history of insomnia in both twin pairs (coincidence of insomnia) and the difference in state of anxiety and trait of anxiety scores. The odds ratios (ORs) for the coincidence of insomnia in monozygotic twins compared to dizygotic twins were analyzed using multiple logistic regression analysis. The estimated values (EV) of the difference of state and trait of anxiety scores were analyzed using a linear regression model. The coincidence of insomnia was not high in monozygotic twins compared to dizygotic twins. The difference in the state of anxiety score was comparable between monozygotic twins and dizygotic twins. However, the difference in anxiety scores was higher in dizygotic twins than in monozygotic twins. The monozygotic twin group did not demonstrate higher coincidence of insomnia or the state of anxiety than the dizygotic twin group. However, the monozygotic twin group indicated higher coincidence of the trait of anxiety than the dizygotic twins. The current results implied the potential contribution of heritable factors for the trait of anxiety.

## 1. Introduction

Anxiety is the most common psychiatric disorder, which was predicted to be approximately 7.3% in the general population worldwide [1,2,3]. The prevalence of anxiety can vary according to the environmental conditions. For instance, the global prevalence of anxiety was estimated to be as high as approximately 33.8% (95% confidence intervals [95% CI] = 29.2–38.7%) among medical students [2]. In addition, its prevalence in the empty-nested old population with the grief that many parents feel when their children move out of the home was estimated to be approximately 41.0% (95% CI = 26–56%) [1]. During the COVID-19 pandemic, the rates of anxiety were calculated to be more than 3 times higher (25%) than those in the prepandemic period (7.3%) [3]. In addition to environmental factors, many studies have suggested that genetic factors may contribute to anxiety [4,5]. Multiple candidate genes for gene–environmental interactions in anxiety have been suggested [5]. Insomnia is one of the associated factors for anxiety, which also has heritability [6,7,8]. In a meta-analysis of twin studies, approximately 40% of heritable factors contributed to the occurrence of insomnia [6]. In addition, it was supposed that there are some overlapping heritable factors of insomnia with anxiety [8]. Thus, both anxiety and insomnia need to be concurrently considered in twin studies.

The genetic contribution to a specific trait can be predicted using a twin study [9,10,11]. One of the most common and basic twin study designs is comparing monozygotic twin pairs with dizygotic twin pairs for the test traits. In this type of study, it is assumed that the genetic concordance of monozygotic twin pairs is 100%, while that of dizygotic twin pairs is 50%. The shared environmental factors are presumed to be equivalent between monozygotic and dizygotic twins. The unshared and unmeasured confounding effects should be considered when interpreting the results of twin studies. Thus, lifestyle factors and socioeconomic factors need to be included when examining the genetic contribution to the traits in twin studies.

This study aimed to evaluate the presence of genetic portions in the trait and state of anxiety and insomnia. To estimate the genetic contribution, monozygotic twins were compared to dizygotic twins for the differences in anxiety and insomnia. In addition, to differentiate the transient state of anxiety from the trait of anxiety, survey questionnaires for both trait and state of anxiety were introduced. This is a novel study comparing differences in anxiety and insomnia in twin cohorts.

## 2. Materials and Methods

### 2.1. Study Population and Data Collection

The ethics committee of Hallym University (2021-03-004) approved the use of these data. The requirement for written informed consent was waived by the Institutional Review Board. This prospective cohort study relied on data from The Korean Genome and Epidemiology Study (KoGES) from 2005 through 2014. A detailed description of these data was described in a previous study [12]. Among the KoGES Consortium, we used KoGES Healthy twin Study (HTS) data consisting of urban residence participants ≥ 20 years old. It consisted of the base data from 2005–2013 and follow-up data from 2008–2014. Among the participants who completed the baseline exam, two-thirds of the participants were followed up, and their medical histories were updated.

### 2.2. Participant Selection

**Figure 1:**
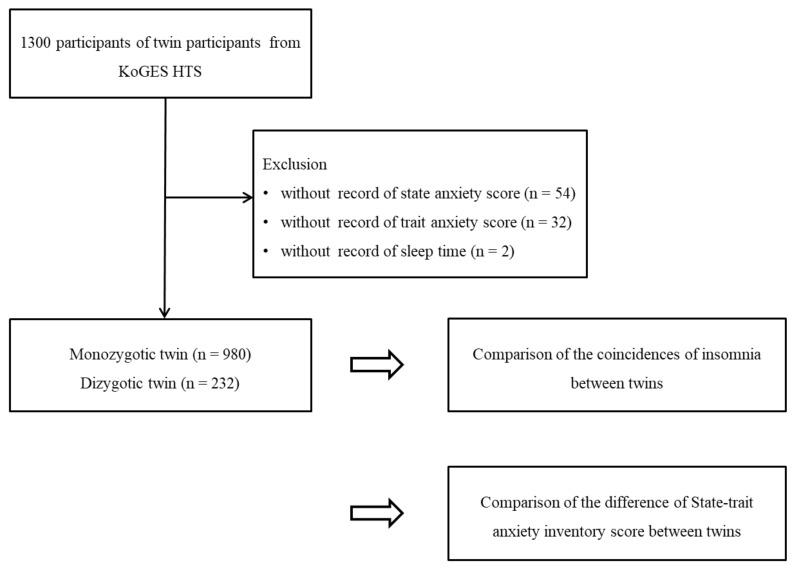
The study design of the present study. The 980 monozygotic twins were compared with 232 dizygotic twins for the coincidences of insomnia between twins and the difference of state and trait of anxiety inventory score between twins.

Among 1300 twin participants, we excluded those who lacked a record of state anxiety score (*n* = 54), trait anxiety score (*n* = 18), and sleep time (*n* = 4). Finally, 980 monozygotic (490 pairs of twins) and 232 dizygotic twin (116 pairs of twins) participants were selected (Figure 1). Then, we analyzed the coincidence of their histories of state-trait anxiety scores and insomnia histories between monozygotic and dizygotic twin participants.

### 2.3. Survey

The participants were asked about their previous histories of insomnia and the state-trait anxiety inventory (STAI) questionnaire by trained interviewees [13]. Insomnia was confirmed if the participants had insomnia histories, answering “yes” instead of “no”. The STAI is composed of the state anxiety score (SAS), which asks about their current feelings (20 questionnaires: 1–4 score), and the trait anxiety score (TAS), which assesses their feelings in general (20 questionnaires: 1–4 score). Scoring was reversed for anxiety-absent items to measure the degree of anxiety [13].

Participants were categorized into four income groups. Based on the household income, the low-income group included the participants whose house income was less than KRW 2 million per month. The middle-income group included the participants whose household income was KRW 2 million to 3 million per month. The middle-high-income group included the participants whose household income was KRW 3 million to 4 million per month. The high-income group included the participants whose house income was KRW 4 million or more per month. Education level was divided into under high school, high school, commercial college—dropped out of college, and graduated from high school college. Marriage status was grouped into unmarried, married, and divorced or other. Physical activity was categorized as hard, moderate, walking time, and sitting time. This was altered based on place of work and movement in the home. Smoking histories were categorized as nonsmoker (<100 cigarettes throughout life), past smoker (quit more than 1 year ago), and current smoker. Alcohol consumption habits were categorized as nondrinker, ≤1 time per month, 2–4 times per month, and ≥2 times per week. Sleep time was calculated as 5/7 weekdays + 2/7 weekend sleep time.

### 2.4. Exposure

Monozygotic twins and dizygotic twins were considered independent variables in this study. All of the participants were twins. There were no triplets, quadruplets or more.

### 2.5. Outcome

We calculated the coincidence of insomnia histories between the matched twin participants. This was categorized as positive–positive, positive–negative, or negative–negative.

We calculated the absolute difference in SAS which implied the current feeling and TAS which represented the feeling in general between the matched twin participants. For example, one twin participant had an SAS of 24, and the other twin participant had an SAS of 21; the absolute difference in SAS was calculated as 3.

### 2.6. Statistical Analyses

The chi-square test (categorical variables) or Wilcoxon rank-sum test (continuous variables) was performed to compare the general characteristics of the participants.

The odds ratios (ORs) for the coincidence of insomnia in monozygotic twins compared to dizygotic twins were calculated using multiple logistic regression analysis.

We calculated the estimated values (EV) with 95% CI of the absolute difference of SAS and TAS. EV was measured as the ‘absolute difference between dizygotic twins’ minus the ‘absolute difference between monozygotic twins’ using a linear regression model.

Crude and adjusted models (age, sex, income, education, marital status, physical activity, obesity, smoking habits, frequency of drinking alcohol, and sleep time) were used.

Two-tailed analyses were conducted, and *p* values less than 0.05 were considered to indicate significance. The results were statistically analyzed using SPSS v. 24.0 (IBM, Armonk, NY, USA).

## 3. Results

The rates of insomnia, state anxiety score, and trait anxiety score were not different between monozygotic and dizygotic twins (all *p* > 0.05, Table 1, Supplementary Tables S1 and S2). The distribution of age groups and sex ratio were different between monozygotic and dizygotic twins (both *p* < 0.05). Other variables such as income level, education level, marital status, level of physical activity, obesity, smoking status, frequency of drinking alcohol, and sleep hours were comparable between the two groups (all *p* > 0.05).

There was no association of the coincidence of insomnia with monozygotic twins compared to dizygotic twins (Table 2). First, the odds for the coincidence of insomnia (positive–positive) or no coincidence of insomnia (negative–negative) in monozygotic twins compared to that of dizygotic twins were estimated. As a result, the odds for the coincidence of insomnia were not high in monozygotic twins (adjusted OR [aOR] = 1.06, 95% CI = 0.52–2.14, *p* = 0.880). Second, the incidence of insomnia (positive–positive or positive–negative insomnia) in monozygotic twins compared to that in dizygotic twins was calculated. The odds for the incidence of insomnia were not high in monozygotic twins (aOR = 1.03, 95% CI = 0.53–2.00, *p* = 0.940).

The differences in state and trait anxiety scores between matched twins were evaluated (Table 3). The difference in state anxiety score was not high in dizygotic twins (EV = 0.18, 95% CI = −0.84–1.20, *p* = 0.728). However, the difference in trait anxiety scores was high in dizygotic twins (EV = 1.88, 95% CI = 0.89–2.86, *p* < 0.001).

## 4. Discussion

The trait of anxiety was more different between dizygotic twins than monozygotic twins. On the other hand, the differences in state of anxiety and insomnia were comparable between dizygotic and monozygotic twins. This study improved previous knowledge on the genetic contribution of anxiety by analyzing twin cohorts with concurrent analysis for insomnia.

A number of prior studies suggested heritable factors for anxiety [14,15,16,17]. Anxiety disorder showed approximately 4–6 times higher odds in family members than in other populations, and heritability was estimated to be approximately 30–50% [18]. However, the candidate genetic loci identified for anxiety disorder varied and overlapped with other psychiatric disorders, such as depression and obsessive-compulsive disorders [19]. In addition, shared environmental factors were suggested to result in the cooccurrence of anxiety traits in twin and singleton studies [20]. In line with this, it was reported that both monozygotic and dizygotic twin pairs showed contagion of anxiety during the 1-year follow-up [16].

The lack of a significantly higher association with the state anxiety score in this study may be attributed to the higher contribution of nonshared environmental factors in the adult population. A meta-analysis demonstrated that the adult population showed a lower genetic contribution to anxiety than the younger population [14]. In addition, the state of anxiety may be more dependent on the specific event or recent distress than heritable factors. To support this assumption, the trait of anxiety was more coincident in monozygotic twins, while the state of anxiety did not show higher coincidence in monozygotic twins than in dizygotic twins in the present study.

Insomnia was not highly coincident in monozygotic twins compared to dizygotic twins in this study. In twin studies based on young populations, the heritability of insomnia has been suggested with mixed results [6,8,21,22]. In a meta-analysis study, the correlation of insomnia was higher in monozygotic twins than in dizygotic twins (0.37, 95% CI = 0.31–0.43 for monozygotic twins and 0.15, 95% CI = 0.12–0.18 for dizygotic twins) [21]. However, other studies suggested weak or questionable heritability of insomnia in multivariable analytic results. Although modest (30.7%) overall heritability of insomnia was estimated in a twin study with an 8- to 16-year-old population, there were no insomnia-specific genetic effects in multivariate analyses [8]. In addition, a meta-analysis indicated high heterogeneity for heritability for sleep quality and sleep duration in twin studies [22]. Thus, unshared environmental factors or other moderators could contribute to insomnia, especially in the adult population.

This study used the KoGES HTS cohort, which regularly validates the data quality by national statisticians. The monozygotic and dizygotic twins were compared, and potential confounders of lifestyle factors, including physical activity, obesity, smoking, alcohol consumption, sleep duration and socioeconomic status, including income level, education level, and marital status, were adjusted. We adjusted income and smoking status because they were acknowledged as associated factors of anxiety [23,24]. However, due to the cross-sectional study design, the causal relationship between monozygotic twins and anxiety cannot be evaluated. In addition, due to the small number of twin pairs with insomnia, the association of the coincidence of insomnia in monozygotic twins was limited in this study. Although numerous variables were adjusted in this study, there may have remained confounders, such as past medical histories of psychiatric disorders. Future studies will be warranted to resolve the current limitations of this study.

## 5. Conclusions

The trait of anxiety coincided more in monozygotic twin pairs than in dizygotic twins. Insomnia and the state of anxiety did not show higher coincidence in monozygotic twins than in dizygotic twins.

## Figures and Tables

**Table 1:** General Characteristics of Participants.

Characteristics		Total Participants		
		Monozygotic Twin	Dizygotic Twin	*p*-Value
Age (years old, *n*, %)				0.004 *
	20–24	6 (0.6)	0 (0)	
	25–29	66 (6.7)	4 (1.7)	
	30–34	332 (33.9)	83 (35.8)	
	35–39	236 (24.1)	63 (27.2)	
	40–44	134 (13.7)	34 (14.7)	
	45–49	112 (11.4)	16 (6.9)	
	50–54	74 (7.6)	22 (9.5)	
	55–59	14 (1.4)	10 (4.3)	
	60–64	4 (0.4)	0 (0)	
	65+	2 (0.2)	0 (0)	
Sex (*n*, %)				0.035 *
	Males	366 (37.3)	104 (44.8)	
	Females	614 (62.7)	128 (55.2)	
Income (*n*, %)				0.955
	<2 million (won)	319 (32.6)	74 (31.9)	
	2 to <3 million (won)	265 (27)	67 (28.9)	
	3 to <4 million (won)	203 (20.7)	47 (20.3)	
	≥4 million (won)	193 (19.7)	44 (19)	
Education (*n*, %)				
	Under high school	116 (11.8)	25 (10.8)	0.928
	Graduated from high school	343 (35)	86 (37.1)	
	Commercial college—Dropped out of college	118 (12)	28 (12.1)	
	Graduated from college	403 (41.1)	93 (40.1)	
Marriage (*n*, %)				0.309
	Unmarried	233 (23.8)	50 (21.6)	
	Married	688 (70.2)	162 (69.8)	
	Divorced or other	59 (6)	20 (8.6)	
Physical Activity				
	Hard (hour/1 week, mean, SD)	3.1 (6.8)	4.5 (9.2)	0.025
	Moderate (hour/1 week, mean, SD)	5.8 (10.4)	6 (9.9)	0.780
	Walk (hour/1 week, mean, SD)	6 (9.3)	6.8 (10.5)	0.283
	Sit (hour/1 week, mean, SD)	40.4 (22.1)	38.7 (20.7)	0.298
Obesity (*n*, %)				0.232
	Underweight (BMI < 18.5)	22 (2.2)	5 (2.2)	
	Normal (BMI ≥ 18.5 to < 23)	482 (49.2)	107 (46.1)	
	Overweight (BMI 23 to < 25)	207 (21.1)	65 (28)	
	Obese I (BMI ≥ 25 to < 30)	238 (24.3)	50 (21.6)	
	Obese II (BMI ≥ 30)	31 (3.2)	5 (2.2)	
Smoking status (*n*, %)				0.173
	Nonsmoker	636 (64.9)	138 (59.5)	
	Past smoker	102 (10.4)	33 (14.2)	
	Current smoker	242 (24.7)	61 (26.3)	
Frequency of drinking alcohol (*n*, %)				0.546
	Nondrinker	278 (28.4)	61 (26.3)	
	≤1 time per month	215 (21.9)	44 (19)	
	2–4 times per month	291 (29.7)	78 (33.6)	
	≥2 times per week	196 (20)	49 (21.1)	
Sleeping hours (*n*, %)				0.270
	≤5 h	52 (5.3)	16 (6.9)	
	6–7 h	583 (59.5)	140 (60.3)	
	8–9 h	320 (32.7)	66 (28.4)	
	≥10 h	25 (2.6)	10 (4.3)	
Insomnia (*n*, %)		29 (3.0)	6 (2.6)	0.760
State anxiety score (mean, SD)		41.4 (10.4)	41.4 (9.7)	0.929
Trait anxiety score (mean, SD)		42.4 (9.6)	43.3 (9.9)	0.222

* Significance at *p* < 0.05. Chi-square test (categorical variables) or Wilcoxon rank-sum test (continuous variables) was performed.

**Table 2:** Analysis of odds ratios with 95% confidence interval of coincidence of insomnia of monozygotic twins compared to dizygotic twins (reference: positive/negative of diseases between twins).

Coincidence of Disease	Monozygotic Twin	Dizygotic Twin	Odds Ratios (95% Confidence Interval)					
	*n* (%)	*n* (%)	Crude	*p*-Value	Model 1 *	*p*-value	Model 2 †	*p*-Value
Insomnia								
Positive–positive or negative–negative	930/980 (94.9)	220/232 (94.8)	0.99 (0.52–1.88)	0.965	1.01 (0.50–2.03)	0.981	1.06 (0.52–2.14)	0.880
Positive–negative	50/980 (5.1)	12/232 (5.2)	1		1		1	
Positive–positive	4/980 (0.4)	0/232 (0.0)	N/A	N/A	N/A	N/A	N/A	N/A
Positive–negative	50/980 (5.1)	12/232 (5.2)	0.99 (0.52–1.89)	0.976	1.00 (0.51–1.94)	0.994	1.03 (0.53–2.00)	0.940
Negative–negative	926/980 (94.5)	220/232 (94.8)	1		1		1	

* Adjusted for age, sex, income, education, marriage status, physical activity, obesity, smoking habit, frequency of drinking alcohol, and sleep time. † Model 1 plus state-trait anxiety score.

**Table 3:** Analysis of estimated values of absolute value of difference between the matched twins (reference: absolute value of difference between monozygotic twin).

Difference of Anxiety	Monozygotic Twin	Dizygotic Twin	Estimated Values of Absolute Difference between Twin (95% CI)					
	Mean (SD)	Mean (SD)	Crude	*p*-Value	Model 1 †	*p*-Value	Model 2 ‡	*p*-Value
State anxiety score	9.1 (7)	9.2 (7.9)	0.11 (−0.92 to 1.14)	0.832	0.16 (−0.86 to 1.18)	0.752	0.18 (−0.84 to 1.20)	0.728
Trait anxiety score	7.9 (6.5)	9.7 (8.4)	1.74 (0.76 to 2.73)	0.001 *	1.87 (0.89 to 2.86)	<0.001 *	1.88 (0.89 to 2.86)	<0.001 *

* Significance at *p* < 0.05. † Adjusted for age, sex, income, education, marriage status, physical activity, obesity, smoking habit, frequency of drinking alcohol, and sleep time. ‡ Model 1 plus state-trait anxiety score.

## Data Availability

Restrictions apply to the availability of these data. Data were obtained from the Korean Genome and Epidemiology Study (KoGES) and are available at https://www.nih.go.kr/contents.es?mid=a50401010100#1 (accessed on 1 January 2022).

## Supplementary Materials

The following supporting information can be downloaded at: https://www.mdpi.com/article/10.3390/jcm11144011/s1, Table S1. State Anxiety Score. Table S2. Trait Anxiety Score.
