# Supplementary material for: Comparison of the Differences in State-Trait Anxiety Inventory Scores and Insomnia Histories between Monozygotic and Dizygotic Twins: A Cross-Sectional Study Using KoGES HTS Data
Source: J Clin Med. 2022 Jul 11;11(14):4011. doi: 10.3390/jcm11144011 (PMC9318741; doi:10.3390/jcm11144011)
Supplement: Supplementary file 1 [file jcm-11-04011-s001.zip › jcm-1796841-supplementary.pdf]

**Table S1** State Anxiety Score

| Characteristics | Total participants | | |
|---|---|---|---|
| | Monozygotic Twin | Dizygotic Twin | P-value |
| I feel calm (n, %) | | | 0.576 |
| Not at all (4) | 73 (7.4) | 23 (9.9) | |
| Somewhat (3) | 313 (31.9) | 73 (31.5) | |
| Moderately so (2) | 461 (47) | 109 (47) | |
| Very much so (1) | 133 (13.6) | 27 (11.6) | |
| I feel secure (n, %) | | | 0.500 |
| Not at all (4) | 115 (11.7) | 27 (11.6) | |
| Somewhat (3) | 317 (32.3) | 83 (35.8) | |
| Moderately so (2) | 427 (43.6) | 101 (43.5) | |
| Very much so (1) | 121 (12.3) | 21 (9.1) | |
| I am tense (n, %) | | | 0.551 |
| Not at all (1) | 367 (37.4) | 91 (39.2) | |
| Somewhat (2) | 415 (42.3) | 103 (44.4) | |
| Moderately so (3) | 160 (16.3) | 29 (12.5) | |
| Very much so (4) | 38 (3.9) | 9 (3.9) | |
| I am regretful (n, %) | | | 0.864 |
| Not at all (1) | 499 (50.9) | 111 (47.8) | |
| Somewhat (2) | 323 (33) | 82 (35.3) | |
| Moderately so (3) | 112 (11.4) | 28 (12.1) | |
| Very much so (4) | 46 (4.7) | 11 (4.7) | |
| I feel at ease (n, %) | | | 0.141 |
| Not at all (4) | 146 (14.9) | 22 (9.5) | |
| Somewhat (3) | 274 (28) | 65 (28) | |
| Moderately so (2) | 416 (42.4) | 112 (48.3) | |
| Very much so (1) | 144 (14.7) | 33 (14.2) | |
| I feel upset (n, %) | | | 0.680 |
| Not at all (1) | 750 (76.5) | 169 (72.8) | |
| Somewhat (2) | 165 (16.8) | 44 (19) | |
| Moderately so (3) | 49 (5) | 14 (6) | |
| Very much so (4) | 16 (1.6) | 5 (2.2) | |
| I am presently worrying over possible misfortunes (n, %) | | | 0.519 |
| Not at all (1) | 448 (45.7) | 109 (47) | |
| Somewhat (2) | 376 (38.4) | 88 (37.9) | |
| Moderately so (3) | 100 (10.2) | 27 (11.6) | |
| Very much so (4) | 56 (5.7) | 8 (3.4) | |
| I feel satisfied (n, %) | | | 0.358 |
| Not at all (4) | 155 (15.8) | 31 (13.4) | |
| Somewhat (3) | 311 (31.7) | 71 (30.6) | |
| Moderately so (2) | 403 (41.1) | 109 (47) | |
| Very much so (1) | 111 (11.3) | 21 (9.1) | |
| I feel frightened (n, %) | | | 0.774 |
| Not at all (1) | 552 (56.3) | 129 (55.6) | |
| Somewhat (2) | 331 (33.8) | 82 (35.3) | |
| Moderately so (3) | 70 (7.1) | 13 (5.6) | |
| Very much so (4) | 27 (2.8) | 8 (3.4) | |
| I feel comfortable (n, %) | | | 0.788 |

| | | | |
|---|---|---|---|
| Not at all (4) | 120 (12.2) | 24 (10.3) | |
| Somewhat (3) | 295 (30.1) | 76 (32.8) | |
| Moderately so (2) | 428 (43.7) | 101 (43.5) | |
| Very much so (1) | 137 (14) | 31 (13.4) | |
| I feel self-confident (n, %) | | | 0.943 |
| Not at all (4) | 104 (10.6) | 27 (11.6) | |
| Somewhat (3) | 339 (34.6) | 81 (34.9) | |
| Moderately so (2) | 434 (44.3) | 102 (44) | |
| Very much so (1) | 103 (10.5) | 22 (9.5) | |
| I feel nervous (n, %) | | | 0.773 |
| Not at all (1) | 404 (41.2) | 93 (40.1) | |
| Somewhat (2) | 434 (44.3) | 99 (42.7) | |
| Moderately so (3) | 107 (10.9) | 30 (12.9) | |
| Very much so (4) | 35 (3.6) | 10 (4.3) | |
| I am jittery (n, %) | | | 0.622 |
| Not at all (1) | 540 (55.1) | 117 (50.4) | |
| Somewhat (2) | 325 (33.2) | 85 (36.6) | |
| Moderately so (3) | 84 (8.6) | 21 (9.1) | |
| Very much so (4) | 31 (3.2) | 9 (3.9) | |
| I feel strained (n, %) | | | 0.294 |
| Not at all (1) | 707 (72.1) | 155 (66.8) | |
| Somewhat (2) | 190 (19.4) | 54 (23.3) | |
| Moderately so (3) | 60 (6.1) | 19 (8.2) | |
| Very much so (4) | 23 (2.3) | 4 (1.7) | |
| I am relaxed (n, %) | | | 0.691 |
| Not at all (4) | 295 (30.1) | 63 (27.2) | |
| Somewhat (3) | 340 (34.7) | 78 (33.6) | |
| Moderately so (2) | 291 (29.7) | 77 (33.2) | |
| Very much so (1) | 54 (5.5) | 14 (6) | |
| I feel content (n, %) | | | 0.210 |
| Not at all (4) | 170 (17.3) | 29 (12.5) | |
| Somewhat (3) | 335 (34.2) | 88 (37.9) | |
| Moderately so (2) | 381 (38.9) | 97 (41.8) | |
| Very much so (1) | 94 (9.6) | 18 (7.8) | |
| I am worried (n, %) | | | 0.054 |
| Not at all (1) | 312 (31.8) | 66 (28.4) | |
| Somewhat (2) | 461 (47) | 126 (54.3) | |
| Moderately so (3) | 150 (15.3) | 35 (15.1) | |
| Very much so (4) | 57 (5.8) | 5 (2.2) | |
| I feel delirious (n, %) | | | 0.277 |
| Not at all (1) | 802 (81.8) | 182 (78.4) | |
| Somewhat (2) | 130 (13.3) | 34 (14.7) | |
| Moderately so (3) | 43 (4.4) | 16 (6.9) | |
| Very much so (4) | 5 (0.5) | 0 (0) | |
| I feel pleasant (n, %) | | | 0.648 |
| Not at all (4) | 182 (18.6) | 37 (15.9) | |
| Somewhat (3) | 330 (33.7) | 76 (32.8) | |
| Moderately so (2) | 400 (40.8) | 99 (42.7) | |
| Very much so (1) | 68 (6.9) | 20 (8.6) | |
| I feel good (n, %) | | | 0.708 |

| | | | |
|---|---|---|---|
| Not at all (4) | 158 (16.1) | 33 (14.2) | |
| Somewhat (3) | 351 (35.8) | 78 (33.6) | |
| Moderately so (2) | 386 (39.4) | 98 (42.2) | |
| Very much so (1) | 85 (8.7) | 23 (9.9) | |

Chi-square test (categorical variables) was performed

**Table S2** Trait Anxiety Score

| Characteristics | Total participants | | |
|---|---|---|---|
| | Monozygotic Twin | Dizygotic Twin | P-value |
| I feel pleasant (n, %) | | | 0.455 |
| Almost never (4) | 85 (8.7) | 27 (11.6) | |
| Sometimes (3) | 366 (37.3) | 80 (34.5) | |
| Often (2) | 468 (47.8) | 108 (46.6) | |
| Almost always (1) | 61 (6.2) | 17 (7.3) | |
| I feel nervous and restless (n, %) | | | 0.101 |
| Almost never (1) | 146 (14.9) | 43 (18.5) | |
| Sometimes (2) | 477 (48.7) | 94 (40.5) | |
| Often (3) | 214 (21.8) | 62 (26.7) | |
| Almost always (4) | 143 (14.6) | 33 (14.2) | |
| I feel like crying (n, %) | | | 0.061 |
| Almost never (1) | 615 (62.8) | 131 (56.5) | |
| Sometimes (2) | 278 (28.4) | 72 (31) | |
| Often (3) | 62 (6.3) | 25 (10.8) | |
| Almost always (4) | 25 (2.6) | 4 (1.7) | |
| I wish I could be as happy as others seem to be (n, %) | | | 0.351 |
| Almost never (1) | 134 (13.7) | 33 (14.2) | |
| Sometimes (2) | 337 (34.4) | 67 (28.9) | |
| Often (3) | 297 (30.3) | 82 (35.3) | |
| Almost always (4) | 212 (21.6) | 50 (21.6) | |
| I fail because I can't make decisions easily (n, %) | | | 0.041* |
| Almost never (1) | 410 (41.8) | 81 (34.9) | |
| Sometimes (2) | 439 (44.8) | 107 (46.1) | |
| Often (3) | 99 (10.1) | 29 (12.5) | |
| Almost always (4) | 32 (3.3) | 15 (6.5) | |
| I feel rested (n, %) | | | 0.450 |
| Almost never (4) | 125 (12.8) | 31 (13.4) | |
| Sometimes (3) | 372 (38) | 77 (33.2) | |
| Often (2) | 414 (42.2) | 110 (47.4) | |
| Almost always (1) | 69 (7) | 14 (6) | |
| I am "calm, cool, and collected" (n, %) | | | 0.859 |
| Almost never (4) | 123 (12.6) | 31 (13.4) | |
| Sometimes (3) | 398 (40.6) | 87 (37.5) | |
| Often (2) | 387 (39.5) | 96 (41.4) | |
| Almost always (1) | 72 (7.3) | 18 (7.8) | |
| I feel that difficulties are piling up so that I cannot overcome them (n, %) | | | 0.344 |
| Almost never (1) | 528 (53.9) | 111 (47.8) | |
| Sometimes (2) | 337 (34.4) | 92 (39.7) | |

| | | | |
|---|---|---|---|
| Often (3) | 90 (9.2) | 21 (9.1) | |
| Almost always (4) | 25 (2.6) | 8 (3.4) | |
| I worry too much over something that really doesn't matter (n, %) | | | |
| Almost never (1) | 273 (27.9) | 61 (26.3) | 0.453 |
| Sometimes (2) | 450 (45.9) | 103 (44.4) | |
| Often (3) | 165 (16.8) | 38 (16.4) | |
| Almost always (4) | 92 (9.4) | 30 (12.9) | |
| I am happy (n, %) | | | 0.722 |
| Almost never (4) | 104 (10.6) | 29 (12.5) | |
| Sometimes (3) | 320 (32.7) | 69 (29.7) | |
| Often (2) | 445 (45.4) | 105 (45.3) | |
| Almost always (1) | 111 (11.3) | 29 (12.5) | |
| I regard anything as hard work (n, %) | | | 0.304 |
| Almost never (1) | 443 (45.2) | 96 (41.4) | |
| Sometimes (2) | 395 (40.3) | 91 (39.2) | |
| Often (3) | 109 (11.1) | 34 (14.7) | |
| Almost always (4) | 33 (3.4) | 11 (4.7) | |
| I lack self-confidence (n, %) | | | 0.164 |
| Almost never (1) | 296 (30.2) | 59 (25.4) | |
| Sometimes (2) | 485 (49.5) | 112 (48.3) | |
| Often (3) | 135 (13.8) | 44 (19) | |
| Almost always (4) | 64 (6.5) | 17 (7.3) | |
| I feel secure (n, %) | | | 0.586 |
| Almost never (4) | 118 (12) | 28 (12.1) | |
| Sometimes (3) | 367 (37.4) | 76 (32.8) | |
| Often (2) | 408 (41.6) | 105 (45.3) | |
| Almost always (1) | 87 (8.9) | 23 (9.9) | |
| I struggle for escaping danger (n, %) | | | 0.510 |
| Almost never (1) | 193 (19.7) | 37 (15.9) | |
| Sometimes (2) | 466 (47.6) | 121 (52.2) | |
| Often (3) | 256 (26.1) | 60 (25.9) | |
| Almost always (4) | 65 (6.6) | 14 (6) | |
| I am depressed (n, %) | | | 0.743 |
| Almost never (1) | 519 (53) | 114 (49.1) | |
| Sometimes (2) | 355 (36.2) | 89 (38.4) | |
| Often (3) | 81 (8.3) | 22 (9.5) | |
| Almost always (4) | 25 (2.6) | 7 (3) | |
| I am content (n, %) | | | 0.942 |
| Almost never (4) | 140 (14.3) | 33 (14.2) | |
| Sometimes (3) | 348 (35.5) | 78 (33.6) | |
| Often (2) | 419 (42.8) | 102 (44) | |
| Almost always (1) | 73 (7.4) | 19 (8.2) | |
| Some unimportant thought runs through my mind and bothers me (n, %) | | | 0.477 |
| Almost never (1) | 292 (29.8) | 73 (31.5) | |
| Sometimes (2) | 475 (48.5) | 100 (43.1) | |
| Often (3) | 149 (15.2) | 42 (18.1) | |
| Almost always (4) | 64 (6.5) | 17 (7.3) | |
| I take disappointments so keenly that I can't put them out of my mind (n, %) | | | 0.325 |
| Almost never (1) | 319 (32.6) | 71 (30.6) | |
| Sometimes (2) | 410 (41.8) | 98 (42.2) | |

| | | | |
|---|---|---|---|
| Often (3) | 154 (15.7) | 46 (19.8) | |
| Almost always (4) | 97 (9.9) | 17 (7.3) | |
| I am a steady person (n, %) | | | 0.079 |
| Almost never (4) | 35 (3.6) | 12 (5.2) | |
| Sometimes (3) | 193 (19.7) | 58 (25) | |
| Often (2) | 472 (48.2) | 111 (47.8) | |
| Almost always (1) | 280 (28.6) | 51 (22) | |
| I get in a state of tension or turmoil as I think over my recent concerns and interests (n, %) | | | 0.808 |
| Almost never (1) | 348 (35.5) | 76 (32.8) | |
| Sometimes (2) | 425 (43.4) | 104 (44.8) | |
| Often (3) | 143 (14.6) | 38 (16.4) | |
| Almost always (4) | 64 (6.5) | 14 (6) | |

* Significance at P < 0.05

Chi-square test (categorical variables) was performed
